# Double-blind controlled trial of lecithinized superoxide dismutase in patients with idiopathic interstitial pneumonia – short term evaluation of safety and tolerability

**DOI:** 10.1186/1471-2466-14-86

**Published:** 2014-05-17

**Authors:** Koichiro Kamio, Arata Azuma, Ken Ohta, Yukihiko Sugiyama, Toshihiro Nukiwa, Shoji Kudoh, Tohru Mizushima

**Affiliations:** 1 Department of Pulmonary Medicine and Oncology, Graduate School of Medicine, Nippon Medical School, 1-1-5, Sendagi, Bunkyo-ku, Tokyo 113-8603, Japan; 2 National Hospital Organization, Tokyo National Hospital, Tokyo, Japan; 3 Division of Pulmonary Medicine, Department of Medicine, Jichi Medical School, Tochigi, Japan; 4 South Miyagi Medical Center, Miyagi, Japan; 5 Fukujuji Hospital, Tokyo, Japan; 6 Department of Analytical Chemistry, Faculty of Pharmacy, Keio University, Tokyo, Japan

**Keywords:** Idiopathic interstitial pneumonia, Idiopathic pulmonary fibrosis, Fibrotic nonspecific interstitial pneumonia, Lecithinized human Cu, Zn-superoxide dismutase, Multicenter double-blind clinical study, Lactate dehydrogenase, Surfactant protein-A

## Abstract

**Background:**

Idiopathic interstitial pneumonias such as idiopathic pulmonary fibrosis or fibrotic nonspecific interstitial pneumonia are irreversible progressive pulmonary diseases that often have fatal outcomes. Although the etiology of idiopathic interstitial pneumonias is not yet fully understood, anti-fibrotic and anti-inflammatory agents have shown limited therapeutic effectiveness. Reactive oxygen species and their cytotoxic effects on the lung epithelial cells have been reported to participate in the pathophysiology of the disease. Because superoxide dismutase catalyzes the detoxification of reactive oxygen species, we developed lecithinized superoxide dismutase for the treatment of patients with idiopathic interstitial pneumonias.

**Methods:**

A multicenter, randomized, placebo-controlled trial was conducted as a pilot study to investigate the safety and effectiveness of 40 or 80 mg lecithinized superoxide dismutase in patients with progressive idiopathic interstitial pneumonias who presented with either idiopathic pulmonary fibrosis or corticosteroid-resistant fibrotic nonspecific interstitial pneumonia and showed arterial oxygen tension compatible with stage III or IV on the Japanese severity grading scale for idiopathic interstitial pneumonias. Before and following infusion of lecithinized superoxide dismutase for 28 days, the primary endpoint of forced vital capacity and the secondary endpoints of lactate dehydrogenase, surfactant protein-A, surfactant protein-D and Krebs von den Lungen-6 levels were measured in the serum.

**Results:**

The primary endpoint of forced vital capacity did not improve significantly in the lecithinized superoxide dismutase groups in comparison with the placebo group. The secondary endpoints of lactate dehydrogenase and surfactant protein-A levels were significantly attenuated by 28 days in the higher-dose (80 mg) group. However, these changes returned to the baseline levels by 56 days after the cessation of lecithinized superoxide dismutase. Adverse events and mortality in the drug-treated groups did not differ from those in the placebo group.

**Conclusions:**

Treatment with lecithinized superoxide dismutase is safe and improves the levels of serum markers such as lactate dehydrogenase and surfactant protein-A in patients with advanced idiopathic interstitial pneumonias with severe respiratory dysfunction. Considering the results of the current study, further investigations into the effects and treatment potential of long-term administration of lecithinized superoxide dismutase may be warranted.

**Trial registration:**

University hospital Medical Information Network (UMIN) clinical trials registry no. 000000752

## Background

Idiopathic interstitial pneumonia (IIP) is a broad term comprising a set of lung diseases, including idiopathic pulmonary fibrosis (IPF), nonspecific interstitial pneumonia (NSIP), cryptogenic organizing pneumonia (COP) and others [1]. While COP has relatively good prognosis among IIPs, IPF and fibrotic NSIP progress irreversibly with most patients eventually dying because of respiratory insufficiency [2,3]. With efforts to develop new treatments, several promising drugs, such as pirfenidone have emerged.

Although the etiology of IIP is not yet fully understood, recent studies have suggested that it is triggered by lung injury and subsequent inflammation. Reactive oxygen species (ROS) and their effects on lung epithelial cells are reported to be involved in the pathophysiology of IIPs [4]. ROS are released from activated leukocytes in response to environmental factors such as air pollutants or cigarette smoke, resulting in further lung injury and inflammation. Furthermore, it has been reported that lung epithelial cells from IPF patients generate higher levels of ROS than those from controls [5]. One study showed that genetic modulation that increased pulmonary levels of ROS resulted in stimulation of bleomycin-induced pulmonary fibrosis [6]. The cellular redox state, determined by the balance between ROS and antioxidant molecules, is considered important in the pathogenesis of IIPs. Therefore, antioxidant molecules are receiving considerable attention as therapeutic candidates for the treatment of IPF.

Superoxide dismutase (SOD) catalyzes the dismutation of superoxide anion to hydrogen peroxide, which is subsequently detoxified to oxygen and water by catalase or glutathione peroxidase [7]. A decreased level of SOD was observed in IPF patients, suggesting that supplementation with SOD could be of therapeutic benefit for patients with IPF [8]. SOD has low affinity to cell or tissue membranes and a half-life of only a few minutes. PC-SOD, a lecithinized human Cu, Zn-superoxide dismutase (SOD) preparation, was developed on the basis of the concepts first proposed by Dr Mizushima Y and colleagues in Japan in the early 1990s [9]. As a result of lecithinization, PC-SOD exerts a high affinity for cell and tissue membranes, maintaining prolonged pharmacological activities in vivo, and ameliorated fibrosis in bleomycin-induced pneumonitis in mice [10]. In humans, the effectiveness and safety of intravenous 40 mg or 80 mg PC-SOD have been demonstrated through administration to patients with ulcerative colitis, in which the involvement of oxidative stress caused by reactive oxygen species is implicated [11].

In this study, we conducted a multicenter, double-blind, placebo-controlled clinical trial of PC-SOD (midismase) to investigate the safety and effectiveness of this agent in patients with advanced IIPs diagnosed as either IPF or corticosteroid-resistant fibrotic nonspecific interstitial pneumonia (srf-NSIP).

## Methods

A multicenter, double-blind, placebo-controlled clinical trial was conducted at 10 medical institutions in Japan for patients with IPF or srf-NSIP. Patients were in agreement with the study procedures and provided informed written consent. The study protocol was approved by the institutional review boards (IRBs) of each participating medical institution (Tohoku University Hospital IRB, Jichi Medical University Hospital IRB, Toho University Sakura Medical Center IRB, Teikyo University Hospital IRB, Nippon Medical School Hospital IRB, JR Tokyo General Hospital IRB, Tosei General Hospital IRB, Tenri Hospital IRB, National Hospital Organization Kinki-Chuo Chest Medical Center IRB, and Saiseikai Kumamoto Hospital IRB). Ongoing safety results were reviewed by an Independent Data Monitoring Committee (IDMC) at 2-month intervals. This clinical trial was registered in the University hospital Medical Information Network (UMIN) clinical trials registry (trial no. 000000752) in June 2007.

Patients with IPF or srf-NSIP as diagnosed by clinical symptoms, chest radiography examination, and high-resolution computed tomography (HRCT) scans were included in this study. Patients with srf-NSIP were defined as having a history of steroid use at doses above 10 mg per day administered for longer than 3 months. In Japan, classification of the severity of IIPs (stages I-IV) has been used for decisions regarding subsidization of medical care. The stages of severity of IIPs are as follows: stage I (partial arterial oxygen concentration ≧80 mmHg at rest), stage II (partial arterial oxygen concentration of 70–80 mmHg at rest), stage III (partial arterial oxygen concentration of 60–70 mmHg at rest), and stage IV (partial arterial oxygen concentration <60 mmHg at rest). Severity should be increased by 1 stage for patients with stage II or stage III disease, if arterial oxygen saturation during a 6-min walk distance test is <90% [12]. This classification is highly correlated with IPF survival [13]. Therefore, eligible patients with stage III or stage IV disease were recruited for this study. Although histopathological diagnosis of NSIP was facilitated by a surgical lung biopsy or transbronchial lung biopsy, IPF was clinically diagnosed in accordance with the ATS/ERS consensus statement [14].

Patients were excluded from this study for the following reasons: complications of asthma, chronic obstructive pulmonary disease, or lung infection; initiation or change in the usage and dosage of corticosteroid treatment in the past 4 weeks; and initiation or alteration in immunosuppressant treatment (ciclosporin, azathioprine, and cyclophosphamide) in the past 2 months.

Patients previously receiving corticosteroids, immunosuppressants, or oxygen inhalation therapy for IIPs were allowed to continue taking these treatments, provided that they did not alter the dosage and/or schedule, whereas no limitation was placed on receiving concomitant therapies for disease complications.

Enrolled patients were equally randomized at the Registration Center by dynamic allocation using a minimization method based on sex, disease severity, and medical institution, to receive either placebo or PC-SOD (40 mg or 80 mg). Each vial of investigational product was masked to ensure double-blind administration. Test agents were intravenously administered by drip infusion over approximately 1 h once daily in the morning for 28 successive days in an inpatient setting.

Patients underwent a forced vital capacity (FVC) test as the primary outcome measurement at baseline, 28 days, and 56 days after starting administration of the investigational drug. Secondary outcome measurements, including percent vital capacity (%VC), total lung capacity (TLC), diffusing capacity for carbon monoxide (%DLCO), and arterial oxygen saturation (SpO_2_), were assessed as indicators of lung function at baseline and on post-treatment days 28 and 56. Serum levels of Krebs von den Lungen-6 (KL-6), surfactant protein (SP)-D, SP-A, and lactate dehydrogenase (LDH) were assessed as markers of interstitial pneumonia at baseline and on days 14, 28, 42, and 56. Each patient's impression of the treatment was assessed by the Borg scale [15].

All items for primary and secondary outcome measurements, except %DLCO, were rated as follows: “improvement”, if improved by >10%; “decline”, if declined by >10%; and unchanged. %DLCO was rated similarly, except that “improvement” was defined as improved by >5%.

All adverse events occurring during the study were thoroughly investigated and their treatment, outcome, and causal relation with the investigational drug were recorded in case report forms. Serious adverse events were defined as fatal, life-threatening, requiring hospitalization or extension of hospitalization, acquisition of permanently lasting or remarkable impairment or dysfunction, or induction of congenital anomaly.

Data on all adverse events, including clinical symptoms and findings, date of incidence, severity level, seriousness, treatment, outcome, and causal relations with the study drug were recorded on case report forms. All adverse events were followed until the lead investigator or secondary investigator concluded that they had subsided or become medically unproblematic. Follow-up data for adverse events were recorded.

### Statistical analysis

The full analysis set (FAS), used for the assessment of efficacy, comprised all patients who completed ≧70% of the investigational drug administration schedule. Safety was investigated in patients who took the investigational drug at least once. Changes in each assessment item during the follow-up period were investigated by a one-way analysis of variance (ANOVA), whereas comparison among the treatment groups was conducted by the least significant difference (LSD) method. Missing data values were replaced by the last observed value of that variable. A significance level of 5% was set for all data.

## Results

### Patients

**Figure 1:**
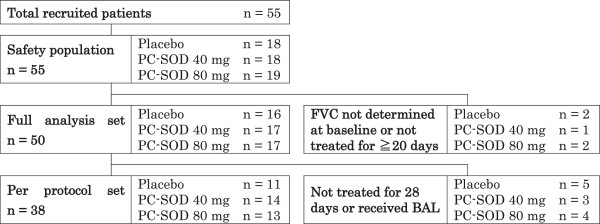
**Disposition of patients.** A total of 55 patients diagnosed with idiopathic pulmonary fibrosis (n = 47) or corticosteroid-resistant fibrotic nonspecific interstitial pneumonia (n = 8) were randomly allocated to receive either a placebo (n = 18), 40 mg of lecithinized superoxide dismutase (PC-SOD) (n = 18), or 80 mg of PC-SOD (n = 19). Of these, 5 patients whose forced vital capacity (FVC) was not determined at baseline or who were not treated with the test agent for ≧20 days were excluded. Therefore, 50 patients were analyzed as a full analysis set. Among these patients, one patient who was not treated by PC-SOD and 11 patients who undertook bronchoalveolar lavage fluid (BAL) analysis were excluded from the per protocol set. While not prohibited in the initial study protocol, performing BAL analysis during the study period was proscribed by the independent data monitoring committee to secure the safety of patients. Therefore, 17 patients (30.9%) were excluded from the study population.

Between June and September 2007, a total of 55 patients (diagnosis of IPF, n = 47; srf-NSIP, n = 8) were randomly allocated to receive either a placebo (n = 18), 40 mg of PC-SOD (n = 18), or 80 mg of PC-SOD (n = 19). Of these, 5 patients (9.1%) whose FVC was not determined at baseline or who were not treated with the test agents for ≧20 days were excluded. Therefore, 16 patients receiving the placebo, 17 patients receiving 40 mg of PC-SOD, and 17 patients receiving 80 mg PC-SOD were analyzed as FAS (Figure 1). The demographic characteristics of these patients were not significantly different among treatment groups, with the exception of disease category (Table 1). Although some patients received corticosteroids and immunosuppressive agents, the frequency of usage was not different between the groups.

**Table 1:** Patient demographics in 3 treatment groups

Parameter*	**Placeo (n = 16)**	**40 mg PC-SOD (n = 17)**	**80 mg PC-SOD (n = 17)**	***P*-value**
Disease category, n (%)				
IPF	16 (100.0)	12 (70.6)	16 (94.1)	0.037
NSIP	0 (0.0)	5 (29.4)	1 (5.9)	
Sex, n (%)				
Male	14 (87.5)	14 (82.4)	14 (82.4)	1.000^#^
Female	2 (12.5)	3 (17.6)	3 (17.6)	
Medical history, n (%)				
No	3 (18.8)	3 (17.6)	6 (35.3)	0.537^#^
Yes	13 (81.3)	14 (82.4)	11 (64.7)	
Concomitant disease, n (%)				
No	0 (0.0)	0 (0.0)	1 (5.9)	1.000^#^
Yes	16 (100.0)	17 (100.0)	16 (94.1)	
Corticosteroid use^**§**^, n (%)				
No	9 (56.2)	5 (29.4)	9 (52.9)	0.286^†^
Yes	7 (43.8)	12 (70.6)	8 (47.1)	
Immunosuppressant use^¶^, n (%)				
No	10 (62.5)	10 (58.8)	12 (70.6)	0.900^†^
Yes	6 (37.5)	7 (41.2)	5 (29.4)	
Age (years)	66.8 ± 8.1	66.4 ± 7.0	67.4 ± 9.0	0.943^††^
Body weight (kg)	65.2 ± 10.6	61.9 ± 10.7	62.1 ± 10.5	0.608^††^
FVC (mL)	2158 ± 706	1819 ± 481	1857 ± 471	0.177^††^
%VC (%)	68.2 ± 17.3	61.5 ± 12.7	59.6 ± 11.2	0.197^††^
TLC (mL)	3113 ± 825	2834 ± 719	2842 ± 667	0.501^††^
%DLCO (%)	32.67 ± 7.95	32.01 ± 16.08	34.88 ± 15.45	0.827^††^
SpO_2_ (%)	94.94 ± 2.21	95.06 ± 2.14	93.76 ± 2.97	0.254^††^
KL-6 (U/mL)	1312.8 ± 473.0	1656.8 ± 932.5	1348.1 ± 555.9	0.290^††^
SP-D (ng/mL)	300.6 ± 200.49	272.53 ± 124.18	314.84 ± 301.20	0.852^††^
SP-A (ng/mL)	90.34 ± 46.99	92.41 ± 43.36	90.58 ± 38.49	0.988^††^
LDH (IU/L)	245.6 ± 63.6	241.8 ± 41.0	287.2 ± 88.4	0.104^††^
Borg scale	3.2 ± 2.1	3.5 ± 2.6	2.8 ± 2.2	0.672^††^

*Parameters other than sex, medical history, concomitant disease, corticosteroid use, and immunosuppressant use are expressed as mean ± SD.

^#^Fisher’s exact test.

^**§**^> 5 mg/day prednisone.

^**†**^Pearson’s *χ*^2^ test.

^¶^An immunosuppressant was chosen from ciclosporin, cyclophosphamide or azathioprine.

^**††**^One-way analysis of variance (ANOVA).

Definition of abbreviations: IPF = idiopathic pulmonary fibrosis; NSIP = nonspecific interstitial pneumonia, PC-SOD = lecithinized superoxide dismutase; FVC = forced vital capacity; VC = vital capacity; TLC = total lung capacity; DLCO = diffusing capacity for carbon monoxide; KL-6 = Krebs von den Lungen-6; SP-D = surfactant protein-D; SP-A = surfactant protein-A; LDH = lactate dehydrogenase.

**Figure 2:**
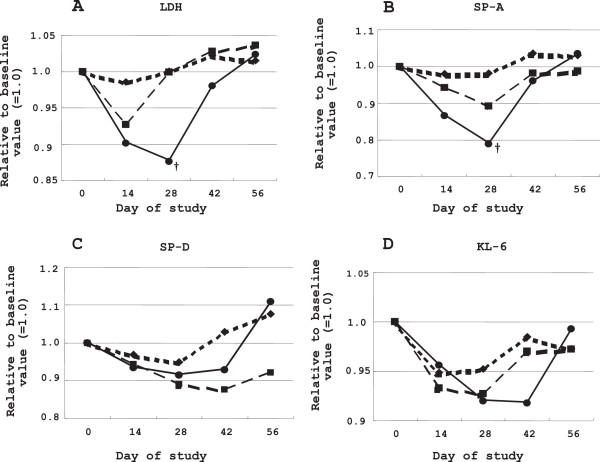
**Time-course of changes in idiopathic interstitial pneumonia markers.** Treatment with the investigational drug was conducted from days 1 to 28. Diamonds, squares, and circles in panels **A**, **B**, **C**, and **D** indicate the placebo, 40 mg lecithinized superoxide dismutase (PC-SOD), and 80 mg PC-SOD, respectively. **(A)** Serum lactate dehydrogenase (LDH) significantly improved compared to the placebo in the 80 mg PC-SOD group on day 28 (^†^*P* < 0.05). Serum LDH returned to the baseline level within 56 days after the discontinuation of PC-SOD. **(B)** Serum surfactant protein (SP)-A significantly improved compared to the placebo in the 80 mg PC-SOD group on day 28 (^†^*P* < 0.05). Serum SP-A returned to the baseline level within 56 days after the discontinuation of PC-SOD. **(C)** Serum SP-D did not improve with PC-SOD treatment. **(D)** Serum Krebs von den Lungen-6 (KL-6) did not improve with PC-SOD treatment. Ordinate: Relative values of serum levels of LDH (A), SP-A **(B)**, SP-D **(C)**, and KL-6 **(D)** to their baseline levels (arbitrarily set as unity). Abscissa: Time (days) after the study was started.

No significant differences in mean change of FVC (the primary efficacy endpoint) from baseline, pulmonary function tests (such as %VC, TLC, %DLCO, and SpO_2_ at rest), and subjects’ impressions (Borg scale) were detected among the 3 groups on days 28 and 56 (Table 2). Conversely, significant improvements of LDH and SP-A were observed in the treatment group receiving the higher-dose of PC-SOD (80 mg) than the placebo group on day 28 (Table 2). Furthermore, these changes were observed in a dose-dependent manner (Table 2). The time-course of changes in interstitial pneumonia markers for each group is depicted in Figure 2. Both LDH and SP-A returned to baseline levels by 56 days after discontinuation of PC-SOD.

**Table 2:** Changes in primary and secondary efficacy variables: days 28 and 56

	**Amount of change**		
**Parameter**	**Placebo (n = 16)**	**40 mg PC-SOD (n = 17)**	**80 mg PC-SOD (n = 17)**
**Day 28**			
FVC, %	2.068 ± 6.539	-2.567 ± 12.175	2.927 ± 11.822
LDH, %	0.0 ± 11.3	-0.1 ± 22.1	-12.4 ± 11.0*
SP-A, %	-1.9 ± 20.8	-10.9 ± 22.3	-21.2 ± 16.9^†^
SP-D, %	-5.1 ± 24.4	-10.9 ± 31.5	-8.6 ± 45.6
KL-6, %	-4.7 ± 14.4	-7.3 ± 16.8	-8.0 ± 18.5
Borg scale	-0.19 ± 0.77	0.26 ± 1.94	0.15 ± 1.41
**Day 56**			
FVC, %	1.127 ± 7.951	-1.993 ± 13.517	0.606 ± 13.716
LDH, %	1.5 ± 14.4	3.6 ± 24.8	2.4 ± 19.3
SP-A, %	3.1 ± 18.1	-1.3 ± 23.8	3.4 ± 19.9
SP-D, %	7.6 ± 41.4	-7.8 ± 35.5	10.9 ± 66.1
KL-6, %	-2.7 ± 17.9	-2.8 ± 18.6	-0.7 ± 21.1
Borg scale	-0.06 ± 1.08	0.06 ± 1.08	0.97 ± 2.24

Mean ± SD. *P < 0.05 vs. placebo, ^†^*P* < 0.01 vs. placebo.

Definition of abbreviations: PC-SOD = lecithinized superoxide dismutase; FVC = forced vital capacity; LDH = lactate dehydrogenase; SP-A = surfactant protein-A; SP-D = surfactant protein-D; KL-6 = Krebs von den Lungen-6.

### Safety

Incidence rates of adverse events (total and those associated with the study medication), a detailed list of serious adverse events, and mortality numbers and rates are displayed in Table 3. Incidence rates of adverse events in the placebo, 40 mg PC-SOD, and 80 mg PC-SOD groups were 83.3, 88.9, and 100.0%, respectively. Those associated directly with PC-SOD were 44.4% for 40 mg PC-SOD and 73.7% for 80 mg PC-SOD. No significant differences were detected among the groups in the incidence rates of adverse events and those associated with PC-SOD. Furthermore, the incidence of serious adverse events was not significantly different among the 3 groups (Table 3).

**Table 3:** Incidence rates of total adverse events and adverse events associated with the study medication, list of serious adverse events, and mortality numbers and rates

	**Placebo (n = 18)**	**40 mg PC-SOD (n = 18)**	*P*-value*	**80 mg PC-SOD (n = 19)**	*P*-value*
**Number of patients with at least 1 adverse event (%)**	15 (83.3)	16 (88.9)	1.000	19 (100.0)	0.105
**Number of patients with at least 1 adverse event directly associated with PC-SOD (%)**	—	8 (44.4)	—	14 (73.7)	0.184^†^
**Serious adverse event, n (%)**					
Myocardial ischemia	0 (0.0)	1 (5.6)	1.000	0 (0.0)	—
Pneumonia	1 (5.6)	0 (0.0)	—	2 (10.5)	1.000
Pneumothorax	0 (0.0)	0 (0.0)	—	2 (10.5)	1.000
Pulmonary embolism	0 (0.0)	1 (5.6)	1.000	0 (0.0)	—
Interstitial lung disease^¶^	3 (16.7)	3 (16.7)	1.000	3 (15.8)	1.000
Pneumomediastinum	0 (0.0)	0 (0.0)	—	1 (5.3)	1.000
Febrile disorders	1 (5.6)	1 (5.6)	1.000	0 (0.0)	—
Acute respiratory failure	0 (0.0)	0 (0.0)	—	1 (5.3)	1.000
**Mortality, n (%)**	3 (16.7)	2 (11.1)		2 (15.8)	

*Fisher’s direct probability test vs. placebo.

^†^Fisher’s direct probability test vs. 40 mg PC-SOD.

^¶^Nine patients were required to stay in the hospital over the study period because of progression of interstitial lung disease.

Definition of abbreviation: PC-SOD = lecithinized superoxide dismutase.

Eight patients died during the study period due to concomitant diseases or acute exacerbation (AE) of IIPs (placebo group, n = 3; 40 mg PC-SOD, n = 2; 80 mg PC-SOD, n = 3) (Table 3). No significant differences in mortality rate were observed among the 3 groups. The cause of death in all cases was AE of interstitial pneumonia (IP), with the exception of one fatality in the high-dose group. AEs of IP occurred from days 22 to 71 after the start of PC-SOD.

## Discussion

Among IIPs, there are devastating diseases such as IPF and f-NSIP that particularly deteriorate respiratory function [16,17]. The pathological process advances gradually and exacerbates, usually resulting in death due to respiratory dysfunction. Thus, therapeutic methods that contribute to ameliorating the prognosis are urgently required.

The primary aim of the present study was to confirm the safety of PC-SOD when used for the treatment of advanced-stage IPF and srf-NSIP. We observed no significant difference in the occurrence of adverse events, and serious adverse events were similar among the active and control groups. During the study, 8 patients died as a result of progression of their original disease or complications. However, no significant difference was detected in the mortality rate among the 3 groups.

Among the causes of death, AE of IP was most frequently observed, and all those who died were patients with IPF. Because drugs such as anti-cancer or anti-tumor necrosis factor-α may exacerbate pre-existing IP [18,19], there was concern that administration of PC-SOD would induce AE of IP. Although AE of IP occurred during the study period, the frequency was similar between the 3 groups. Therefore, it is unlikely that PC-SOD accelerated the exacerbation of IP. Moreover, we previously administered 80 mg of PC-SOD repeatedly for 7 days in a phase I study conducted in healthy volunteers, and safely repeated the dosing over 14 days in a phase II study performed in patients with ulcerative colitis who did not show acute lung injury [11]. Thus, there are no particular safety concerns regarding the use of 40–80 mg PC-SOD in patients with IPF and srf-NSIP.

In the current study, 7 out of 55 (12.3%) patients experienced AE of IP. This frequency appears to be higher than that in a previous study in which the 2-yr frequency of AE of IP was reported to be 9.6% [20]. We assumed that the high frequency of AE of IP in this study was due to the current inclusion criteria which aimed to recruit more severely ill patients with stage III or IV disease, as classified on the Japanese severity scale of IIPs. This inclusion criteria was considered appropriate because PC-SOD was administered to patients intravenously, requiring the patients to be hospitalized during the trial period. Moreover, patients with severe disease with relatively prominent clinical symptoms were deemed suitable for the evaluation of the efficacy and safety of the investigational product.

Although changes in FVC was the primary outcome measurement in this study, there was no significant difference between the study drug groups and the placebo group. Changes in FVC or VC are considered to be important indicators in prognosis of IPF [21-24], and both pirfenidone and nintedanib (BIBF 1120), which have anti-fibrotic properties, have been reported to ameliorate the decline of VC and FVC, respectively [25-27]. Most of these studies see changes in FVC within 6 to 12 months. The 56-day observation period in this study appears to be too short to detect a significant difference. Considering this, longer-term continuous administration of the study drug is required for future study.

Despite the absence of significant changes in FVC, 80 mg PC-SOD significantly improved the levels of biological markers such as serum LDH and SP-A, compared to the levels in the placebo group at 28 days after drug administration. Both LDH and SP-A have been reported to increase in patients with IIPs [28,29]. These increase in parallel with an extended radiological display of a ground-glass appearance, and are important as parameters to predict early mortality of IPF patients [30,31]. Although LDH is a marker that lacks organ specificity, baseline levels in our IIP patients were higher than 240 IU/L, suggesting that the LDH was released from pulmonary tissue cells. Thus, the decrease in the serum LDH level following injection of PC-SOD was interpreted as an indication of the drug’s favorable effects on pulmonary tissue cells. In contrast to LDH, SP-A is secreted mainly from type II pneumocytes, and the attenuation of SP-A after the administration of PC-SOD appears to reflect the essential effect of this agent, although improvement of survival rate was not observed in the current study. It is important to note that both LDH and SP-A reverted to original levels by termination of the treatment on day 56. These results suggest that continuous treatment with PC-SOD may be required to elicit a clinical benefit.

In this study, PC-SOD was administered intravenously for 28 consecutive days. However, in consideration of the quality of life of patients, daily intravenous administration of the drug is not an optimal treatment approach. To resolve this problem, we have developed a method of PC-SOD administration by inhalation and have demonstrated that this procedure is effective against bleomycin-induced pulmonary fibrosis in mice [32,33]. Future studies of PC-SOD administered by inhalation for IPF patients are planned.

We performed the analysis using FAS excluding patients whose baseline FVC was not determined or who were not treated for ≧20 days. Among the 5 patients who were excluded from FAS for being treated for less than 20 days, one turned out to be in violation of the inclusion criteria (absence of FVC before randomization to the study). The use of FAS is considered to be acceptable since it provides estimates of treatment effects which are likely to mirror those observed in subsequent practice. We also performed the analysis using the per protocol set (PPS). Since this analysis yielded similar results to FAS, the PPS findings were not presented. We believe that any potential bias regarding the treatment outcome has been eliminated in this study.

In the current study, we tested PC-SOD in a population of patients with different diseases, including IPF and NSIP. While including patients with different diseases in a trial may appear unusual, the prognosis of NSIP is variable and some patients progress to end-stage fibrosis and eventually die of the disease [1,34]. Five-year survival rate of fibrotic NSIP has been reported to be 74 or 82.3% in previously published studies [35,36]. Moreover, oxidative stress is speculated to be involved in the pathogenesis of NSIP [5]. Therefore, we recruited patients with NSIP that did not respond to steroid, as well as those with IPF.

## Conclusions

PC-SOD in patients with moderate-to-severe IIP (IPF and srf-NSIP) is well tolerated and may be a promising agent for the improvement of biomarker levels. The higher dose (80 mg) of PC-SOD is more effective than the lower dose (40 mg) in reducing biomarker levels. A larger-scale study conducted over a longer period is needed to confirm the benefits to pulmonary function and survival rate in patients with advanced IPF and srf-NSIP.

## Competing interests

Financial support for this clinical study and test agents were provided by LTT Biopharma Co., Ltd.

## Authors’ contributions

KK contributed to study design, collection of data, analysis and interpretation of data, and writing the draft. AA contributed to study design, collection of data, analysis and interpretation of data, and editing the draft. KO, YS, TN, and SK contributed to study design, collection of data, and analysis and interpretation of data. TM contributed to analysis and interpretation of data, editing the draft, and acquisition of funding. All authors read and approved the final manuscript.

## Pre-publication history

The pre-publication history for this paper can be accessed here:

http://www.biomedcentral.com/1471-2466/14/86/prepub
